# Screening auf Frühgeborenenretinopathie – die wichtigsten Änderungen in der neuen deutschen Leitlinie 2020

**DOI:** 10.1007/s00347-021-01393-6

**Published:** 2021-04-30

**Authors:** Jeany Q. Li, Ulrich Kellner, Birgit Lorenz, Andreas Stahl, Tim U. Krohne

**Affiliations:** 1 Universität zu Köln, Zentrum für Augenheilkunde, Medizinische Fakultät und Uniklinik Köln, Kerpener Str. 62, 50937 Köln, Deutschland; 2 AugenZentrum Siegburg, MVZ Augenärztliches Diagnostik- und Therapiecentrum Siegburg GmbH, Siegburg, Deutschland; 3 Universitäts-Augenklinik, Universität Bonn, Bonn, Deutschland; 4 Klinik und Poliklinik für Augenheilkunde, Justus-Liebig-Universität Gießen, Gießen, Deutschland; 5 Klinik und Poliklinik für Augenheilkunde, Universitätsmedizin Greifswald, Greifswald, Deutschland

**Keywords:** Gestationsalter, Intravitreale Injektion, Ranibizumab, Bevacizumab, Laserkoagulation, Gestational age, Intravitreal injection, Ranibizumab, Bevacizumab, Laser coagulation

## Abstract

**Hintergrund:**

Durch Verbesserungen in der neonatologischen Versorgung von Frühgeborenen und die Entwicklung neuer Behandlungsmöglichkeiten der Frühgeborenenretinopathie („retinopathy of prematurity“ [ROP]) haben sich die Anforderungen an das ROP-Screening seit der Veröffentlichung der letzten Fassung der deutschen Leitlinie zum ROP-Screening im Jahr 2008 verändert. Auf Grundlage aktueller Studiendaten wurde die Leitlinie in 2020 grundlegend überarbeitet und in einer aktualisierten Fassung veröffentlicht.

**Ziel:**

Dieser Artikel fasst die wichtigsten Änderungen in der neuen Leitlinie zusammen.

**Ergebnisse:**

Die Altersgrenze für einen Screeningeinschluss wurde für Kinder ohne zusätzliche Risikofaktoren auf ein Gestationsalter von unter 31 Wochen gesenkt. Die Mindestdauer für eine Sauerstoffsupplementation, die einen Einschluss in das Screening bei Frühgeborenen erforderlich macht, wurde auf über 5 Tage angehoben. Eine Behandlung bei ROP in Zone II kann nun schon bei jedem Stadium 3 mit Plus-Symptomatik unabhängig von der Anzahl der betroffenen Uhrzeiten erfolgen. Für die Nachkontrollen nach Anti-VEGF („vascular endothelial growth factor“)-Therapie wurden Kriterien zur Frequenz und Dauer definiert. Das verbindliche Dokument für diese und weitere neue Empfehlungen ist die Leitlinie selber.

**Schlussfolgerungen:**

Die Empfehlungen der Leitlinie ermöglichen eine zuverlässige Identifikation von Kindern mit ROP-Risiko für den Einschluss in das Screening und eine rechtzeitige Erkennung fortgeschrittener Krankheitsstadien für die Therapieeinleitung, um so Erblindung durch ROP zu verhindern.

## Abstract (en)

**Background:**

Due to improvements in neonatal care of premature infants and the development of novel treatment options for retinopathy of prematurity (ROP), the requirements for screening for ROP have changed since publication of the last version of the German ROP screening guideline in 2008. Based on results of recent studies, the guideline has been extensively revised in 2020 and published in an updated version.

**Objective:**

This article summarizes the most important changes in the new guideline.

**Results:**

The age limit for screening inclusion was lowered to a gestational age of below 31 weeks for infants without additional risk factors. The minimum duration of oxygen supplementation necessitating screening inclusion in preterm infants was increased to more than 5 days. Treatment for ROP in zone II can now be given at any stage 3 with plus disease, regardless of the number of clock hours affected. Criteria for the frequency and duration have been defined for follow-up examinations after anti-vascular endothelial growth factor (VEGF) treatment. The binding document for these and other new recommendations is the guideline itself.

**Conclusion:**

The guideline recommendations enable a reliable identification of infants at risk for ROP for screening inclusion and a timely detection of advanced disease stages for treatment initiation, thus preventing blindness from ROP.

Das augenärztliche Screening auf Frühgeborenenretinopathie („retinopathy of prematurity“ [ROP]) hat sich in den letzten Jahrzehnten als hochwirksame Maßnahme bewährt, um eine rechtzeitige Therapie der betroffenen Kinder zu ermöglichen und dadurch das Risiko für eine hochgradige Sehbehinderung oder Erblindung durch die ROP deutlich zu reduzieren. Mit Verbesserungen in der neonatologischen Versorgung der Frühgeborenen haben sich auch die Screeningerfordernisse in den vergangenen Jahren wesentlich gewandelt [1]. Gleichzeitig wurde durch die Einführung der Anti-VEGF(„vascular endothelial growth factor“)-Therapie die Behandlung der ROP revolutioniert. So erfolgten 2016 bereits 53 % aller ROP-Behandlungen in Deutschland mittels Anti-VEGF-Therapie [2].

Vor diesem Hintergrund wurde die deutsche Leitlinie zum ROP-Screening in der Version von 2008 [3] sowie die Stellungnahme zur Anti-VEGF-Therapie der ROP in der bisher gültigen Version von 2011 [4] überarbeitet und beide Dokumente 2020 in einer aktualisierten Fassung veröffentlicht. Während die Stellungnahme zur ROP-Therapie bereits in *Der Ophthalmologe* erschienen ist [5], ist die Leitlinie zum ROP-Screening für einen Abdruck zu umfangreich und erscheint deshalb in diesem Journal ausschließlich online [6]. In dieser Übersicht werden die wichtigsten Änderungen in der neuen Leitlinie zusammengefasst, wobei jedoch für jeden Augenarzt, der Frühgeborene betreut, die Kenntnis der vollständigen Leitlinie mit ihrer detaillierten Darstellung der klinischen Empfehlungen und der ihnen zugrunde liegenden wissenschaftlichen Daten erforderlich ist.

## Wer benötigt ein Screening?

In das ROP-Screening sollen alle Frühgeborenen aufgenommen werden, die ein erhöhtes Risiko für die Entwicklung einer therapiebedürftigen Retinopathie besitzen. Gleichzeitig sollen die Screeningkriterien so spezifisch wie möglich sein, um unnötige Untersuchungen zu vermeiden. In Deutschland lag die empfohlene Altersobergrenze für einen Screeningeinschluss in der Leitlinie von 2008 bei einem Gestationsalter von < 32 + 0 Schwangerschaftswochen (SSW) [3], sofern keine weiteren Risikofaktoren vorlagen, und damit höher als in den meisten anderen Industrieländern (Tab. 1; [7–12]). In einer aktuellen Auswertung des Schwedischen Nationalen ROP-Registers wies von 440 wegen ROP behandelten Kindern keines ein Gestationsalter von 30 + 0 SSW oder darüber auf [11]. Für Deutschland zeigte eine aktuelle Auswertung von 1505 wegen ROP behandelten Kindern ein Gestationsalter von 30 + 0 SSW oder darüber bei 1,4 % [13]. Eine Analyse von Daten des Deutschen Retina.net-ROP-Registers (www.rop-register.de) bestätigte den Anteil von 1,4 % und ergab zudem, dass alle diese behandelten Kinder mit einem Gestationsalter von 30 + 0 SSW oder darüber noch unabhängige Risikofaktoren wie eine langfristige Sauerstoffsupplementation oder relevante Begleiterkrankungen aufwiesen [1]. Aufgrund dieser Datenlage wurde in der Neufassung der Leitlinie die obere Altersgrenze für einen Einschluss ins ROP-Screening für Kinder ohne zusätzliche Risikofaktoren von einem Gestationsalter von bisher 32 + 0 Wochen auf nun 31 + 0 SSW herabgesetzt. Dadurch kann vielen Kindern eine unnötige Screeninguntersuchung erspart werden, ohne dabei das Risiko zu erhöhen, Kinder mit behandlungsbedürftiger ROP zu übersehen.

**Table 1:** 

Land	Gestationsaltersgrenze für obligates ROP-Screening	Zusatzkriterien für ROP-Screening	Publikationsjahr der Leitlinie	Referenz
Großbritannien	< 31 SSW („must“) < 32 SSW („should“)	< 1251 g („must“) < 1501 g („should“)	2008	[7]
Schweden	< 30 SSW	–	2020	[11]
Niederlande	< 30 SSW	< 1250 g Bei Risikofaktoren 32 SSW bzw. < 1500 g	2013	[8]
Kanada	< 31 SSW	Bei Risikofaktoren höheres Gestationsalter	2006	[9]
USA	< 31 SSW	< 1500 g Bei Risikofaktoren höheres Gestationsalter	2018	[12]

*ROP* Frühgeborenenretinopathie („retinopathy of prematurity“), *SSW* Schwangerschaftswoche

Wie bisher besteht eine Indikation zum Screening bei Frühgeborenen mit einem höheren Gestationsalter, wenn Risikofaktoren für die Entwicklung einer ROP vorliegen. Bekanntermaßen stellt eine längerfristige Sauerstoffsupplementation einen solchen Risikofaktor dar. Die bisherige Grenze zur Einleitung eines ROP-Screenings von über 3 Tagen Sauerstoffgabe wurde in der neuen Fassung auf über 5 Tage heraufgesetzt. Neu ist außerdem die Aufnahme einer extrakorporalen Membranoxygenierung (ECMO) als Indikation zum Screening von Frühgeborenen unabhängig von Gestationsalter und Dauer der Behandlung, da bei solchen Kindern teils schwere Stadien einer ROP festgestellt wurden [14]. Ebenfalls neu wurden relevante Begleiterkrankungen wie eine schwere nekrotisierende Enterokolitis, bronchopulmonale Dysplasien, Sepsis oder transfusionsbedürftige Anämien in der rezenten Literatur als Risikofaktoren beschrieben [15] und in Leitlinie als neue Kriterien zur Einleitung eines Screenings unabhängig vom Gestationsalter aufgenommen. Alle weiteren Empfehlungen zu den Kriterien für die Aufnahme ins Screening und dem Zeitpunkt für den Beginn des Screenings sind weitgehend unverändert geblieben und können in der neuen Leitlinie nachgelesen werden [6].

## Wie häufig muss das Screening erfolgen?

**Figure 1:**
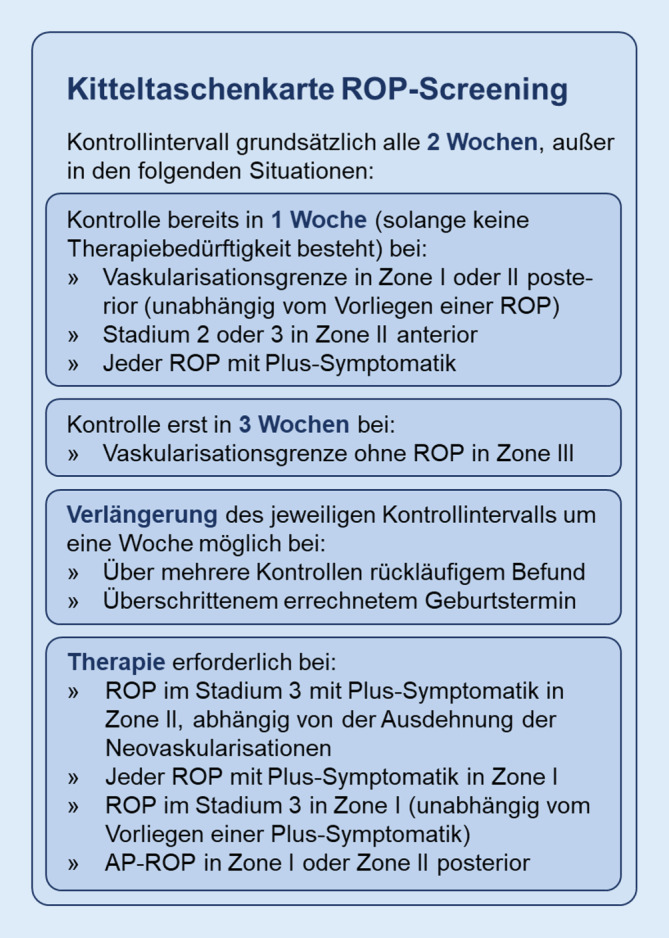

Die Intervalle der Screeninguntersuchungen richten sich nach Stadium und Zone der ROP, wie sie in der *International Classification for Retinopathy of Prematurity* (ICROP) definiert sind [16]. Die von der neuen Leitlinie empfohlenen Screeningintervalle wurden zur besseren Übersicht in Abb. 1 tabellarisch zusammengefasst. Grundsätzlich gilt auch in der neuen Leitlinie ein Screeningintervall von 2 Wochen, und eine Reduktion auf einen 1‑wöchentlichen Kontrollabstand erfolgt weiterhin bei einer Vaskularisationsgrenze in Zone I oder posteriorer Zone II oder einer Plus-Symptomatik. Modifiziert wurde die neue Leitlinie in Bezug auf die ROP in Stadium 2 oder 3 in Zone II: Während hier in posteriorer Zone II weiterhin 1‑wöchentliche Kontrollintervalle empfohlen werden, sind in anteriorer Zone II jetzt 2‑wöchentliche Kontrollen ausreichend. Dabei ist die Grenze zwischen posteriorer und anteriorer Zone II als Kreis um die Papille mit dem Radius des 3‑fachen Abstands von Papille zu Fovea definiert [6]. Neu ist auch das verlängerte Kontrollintervall von 3 Wochen bei Vaskularisationsgrenze in Zone III. Unverändert geblieben sind die Empfehlungen zum Beginn und zum Abschluss der Screeninguntersuchungen, die sich zusammen mit weiteren Empfehlungen zur Wahl der Screeningintervalle in der neuen Leitlinie finden [6].

## Wer muss behandelt werden?

**Figure 2:**
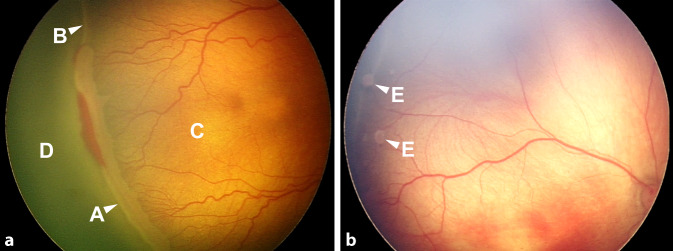

Die Therapieindikation bei der ROP wird basierend auf dem vorliegenden Krankheitsstadium gestellt. Eine Auflistung der Therapiekriterien der neuen Leitlinie findet sich in Abb. 1, ein exemplarischer Befund einer ROP im Stadium 3 in Abb. 2. Während die deutsche Leitlinie eine Therapie in Zone II erst bei einem Stadium 3 mit Plus-Symptomatik vorsieht, wird in anderen nationalen Leitlinien wie der US-amerikanischen eine Behandlung schon früher, nämlich bei Vorliegen eines Stadiums 2 mit Plus-Symptomatik empfohlen [12]. In der neuen deutschen Leitlinie wurden die Therapieindikationen nun offener formuliert und ausgeweitet, um eine individuellere Anpassung der Therapie an die Bedürfnisse des einzelnen Kindes zu ermöglichen. Weiterhin gilt dabei die Behandlungsempfehlung für eine ROP im Stadium 3 mit Plus-Symptomatik in Zone II bei Proliferationen in mehr als 5 zusammenhängenden oder 8 unzusammenhängenden Uhrzeiten. Neu ist, dass jetzt auch schon bei Proliferationen über weniger als die genannte Anzahl von Uhrzeiten eine Therapie möglich ist, wenn dies dem Behandler sinnvoll erscheint. Weiterhin ist eine Behandlung bei ROP in Zone I in jedem Stadium mit Plus-Symptomatik und im Stadium 3 ohne Plus-Symptomatik sowie bei aggressiv posterioren ROP (AP-ROP) in Zone I oder in posteriorer Zone II indiziert [17]. In der neuen Leitlinie sind weitere Details zu den genannten Empfehlungen sowie zusätzliche Kriterien zur Behandlungsindikation aufgeführt [6]. Zur Durchführung der Anti-VEGF-Therapie bei der ROP finden sich in der aktuellen Stellungnahme der Fachgesellschaften zu diesem Thema detaillierte Empfehlungen [5].

## Wie lange muss nach Therapie nachkontrolliert werden?

Mehrere Studien belegen, dass behandlungsbedürftige Krankheitsrezidive nach Anti-VEGF-Therapie noch deutlich später auftreten können als nach Laserbehandlung und deshalb auch die Nachkontrollen nach Anti-VEGF-Therapie sehr viel langfristiger erfolgen müssen [18, 19]. In der BEAT-ROP-Studie mit einer maximalen Nachbeobachtungszeit bis zu einem postmenstruellen Alter von 54 Wochen traten Rezidive nach Bevacizumab-Therapie durchschnittlich nach 16,0 Wochen auf, verglichen mit 6,2 Wochen nach Lasertherapie [20]. Im Anschluss an die Studie fand eine langfristige Nachbeobachtung von 241 mit Bevacizumab behandelten Kindern über durchschnittlich 2,5 Jahre statt [19]. Die Inzidenz des Wiederauftretens eines therapiebedürftigen Stadiums in dieser Kohorte lag bei 8,3 %, wobei das größte Risiko bei Vorliegen einer AP-ROP, einer langen Hospitalisationsdauer oder einem geringeren Geburtsgewicht beschrieben wurde. Im Mittel traten die Rezidive in einem postmenstruellen Alter von 51,2 ± 4,6 Wochen bzw. einem Intervall von 16,2 ± 4,4 Wochen nach der letzten Injektion auf. Andere Studien beschreiben das Auftreten später behandlungsbedürftiger Rezidive in Einzelfällen sogar noch bis zu einem postmenstruellen Alter von 69 Wochen bzw. einem Intervall von 35 Wochen nach der letzten Injektion [21–23]. Daher sind nach Anti-VEGF-Therapie Kontrolluntersuchungen in der Regel noch bis weit über den errechneten Geburtstermin hinaus erforderlich. Eine Laserbehandlung verbliebener avaskulärer Areale nach Anti-VEGF-Therapie ist eine Option, wenn eine vollständige Vaskularisierung ausbleibt [24]. Jedoch wurden auch Spätrezidive trotz erfolgter sekundärer Laserkoagulation beschrieben, möglicherweise aufgrund einer abnormen retinalen Perfusion nach Anti-VEGF-Therapie [25].

Die neue Leitlinie definiert erstmalig Frequenz und Dauer der erforderlichen Nachkontrollen nach erfolgter Anti-VEGF-Therapie. Demzufolge sollen die Kontrollintervalle nach Therapie nach denselben Kriterien gewählt werden, wie sie die Leitlinie für die Screeninguntersuchungen vor Therapie definiert. Die Nachkontrollen nach Anti-VEGF-Therapie können beendet werden, sobald sich die Aktivitätszeichen der ROP (z. B. Plus-Symptomatik, aktive Proliferationen, aktive Traktionen) zurückgebildet haben und zusätzlich entweder die Netzhaut vollständig ausvaskularisiert ist oder noch verbleibende periphere avaskuläre Netzhautareale mit einer Lasertherapie vollständig behandelt wurden oder über mehrere Monate hinweg ein Befund ohne pathologische Gefäßaktivität beobachtet wurde. Nach erfolgter Lasertherapie mit ausreichender Ablation aller avaskulären Areale können die Nachkontrollen beendet werden, wenn ebenfalls ein stabiler Befund mit Rückbildung der Aktivitätszeichen der ROP eingetreten ist. Weitere Empfehlungen zu den Nachkontrollen finden sich in der neuen Leitlinie [6]. Die Stellungnahme der Fachgesellschaften zur Anti-VEGF-Therapie der ROP gibt zudem detaillierte Empfehlungen zur Wiederbehandlung bei unzureichendem Therapieansprechen oder bei Rezidiven [5].

Um die Kontinuität der Nachkontrollen auch bei einem Arztwechsel, beispielsweise bei Entlassung des Kindes aus dem Krankenhaus, zu gewährleisten, soll eine schriftliche Übergabe aller relevanten klinischen Daten wie erfolgte Behandlungen, bisherige und aktuelle Befunde und empfohlener Zeitpunkt der nächsten Kontrolle erfolgen. In der neuen Leitlinie wurde ein ROP-Pass entwickelt, der zu diesem Zweck verwendet werden und in das Untersuchungsheft des Kindes eingelegt werden kann (https://www.dog.org/wp-content/uploads/2013/03/ROP-Pass_Version-3_4_FORMULAR.pdf).

## Fazit für die Praxis

- Eine regelmäßige Evaluation und Anpassung der Screeningkriterien ist notwendig, um frühgeborenen Kindern ohne ROP-Risiko unnötige Screeninguntersuchungen zu ersparen. Auf Grundlage aktueller Studiendaten zur ROP-Inzidenz bei reiferen Frühgeborenen wird in der neuen Leitlinie zum ROP-Screening die obere Altersgrenze für den Einschluss ins Screening abgesenkt.
- Die Indikationskriterien zur Therapie der ROP werden in der neuen Leitlinie offener formuliert, um dem Behandler mehr Freiheit zur Anpassung der Therapie an den individuellen Patienten zu ermöglichen.
- Neben der Laserkoagulation steht nun auch die Anti-VEGF-Therapie als hochwirksame Behandlungsoption für die ROP zur Verfügung. Eine Herausforderung stellen die möglichen Spätrezidive und die damit verbundenen langfristigen Nachkontrollen nach Anti-VEGF-Therapie dar. Die neue Leitlinie gibt erstmals Empfehlungen zu Frequenz und Dauer der Nachkontrollen nach Therapie.
